# Midwifery Students’ Knowledge and Need of Education Toward Oral Diseases in France

**DOI:** 10.1155/ijod/5058682

**Published:** 2026-04-24

**Authors:** Aikaterini Vasileiou, Lucie Brillaux, Catherine Petit, Pierre-Yves Gegout, Olivier Huck

**Affiliations:** ^1^ Dental Faculty, University of Strasbourg, Strasbourg, France, unistra.fr; ^2^ University Hospital, Strasbourg, France, chru-strasbourg.fr

**Keywords:** gingivitis, midwifery, oral health, periodontal diseases, periodontitis, pregnancy

## Abstract

**Introduction:**

Hormonal and immunological changes during pregnancy can affect oral health, especially periodontal conditions, which are linked to an increased risk of adverse pregnancy outcomes. This study aimed to evaluate the knowledge of midwifery students regarding oral diseases and their prevention during pregnancy.

**Materials and Methods:**

A self‐administered, structured questionnaire was sent to French midwifery students. It comprised 31 questions covering the demographic characteristics of the participants, knowledge of participants regarding the link between oral health and pregnancy, the consideration of oral health during their studies, and their clinical experience related to oral health in pregnant women. Descriptive analyses and univariate analyses were conducted.

**Results:**

A total of 420 students completed the questionnaire during the year 2024. Although 95% of those students acknowledged the importance of oral health during pregnancy, only 67% were aware of its link to adverse outcomes. Overall, 51% of the participants were aware of periodontitis, and only 8% recognized gingival diseases as a potential risk for pregnancy complications. Overall, 38% of the students considered themselves as insufficiently prepared to offer practical oral health advice, with only 2% providing regular counseling. There was a significant discrepancy in education toward periodontal diseases, with 43% of students reporting having lessons on the subject during their studies, mostly for less than 2 h.

**Conclusion:**

The level of students’ knowledge and practice regarding oral health was not sufficient. Curricula improvements and increased clinical experiences are essential to enhance students’ ability to assess oral health and provide preventive care to pregnant women. Future research should evaluate the effectiveness of these interventions.

## 1. Introduction

Pregnancy is associated with numerous hormonal, immunological, and behavioral changes from conception to delivery. Such changes are linked to potential modifications of oral health status [1]. Previous studies showed a lack of oral health education among healthcare professionals, particularly obstetricians and gynecologists, despite maternal oral health’s recognized impact on pregnancy outcomes. These findings emphasize the need for a multidisciplinary approach to improve healthcare providers’ awareness and management of oral health during pregnancy [2]. Pregnant women often experience increased gingival inflammation due to modifications in estrogen and progesterone levels [3]. Gingival bleeding is a common complaint among pregnant women, as observed in a large German cohort, where 45.4% of individuals reported it [4].

The association between pregnancy and maternal periodontal diseases has been extensively studied over the last two decades. Indeed, the link between maternal periodontitis and adverse pregnancy outcomes has been observed, identifying periodontitis as a risk factor for preterm birth, low birth weight, preeclampsia, and preterm low birth weight [5]. Epidemiological findings have highlighted that preterm birth rates range from 5% to 9% in Europe and up to 18% in Africa. Despite advancements, nearly 50% of preterm birth causes remain unexplained, underscoring the importance of addressing periodontal diseases [6]. Furthermore, findings from a related study have emphasized that periodontal treatment during pregnancy may reduce the risk of such complications by up to 50% [7].

Nevertheless, it is important to mention that oral interventions, such as steps 1 and 2 of periodontal therapy [8], are considered safe and effective in preventing and treating periodontal diseases or exacerbations during pregnancy [9]. Such steps of periodontal therapy consist of oral hygiene education, professional mechanical plaque removal (PMPR), risk factor control, supragingival biofilm control, and, when indicated, subgingival debridement [8]. However, several studies have shown that there is a persistent belief that oral care may be unfavorable during pregnancy. This may be partly due to the lack of knowledge and misleading information on social networks [10]. Research suggests that healthcare professionals’ attitudes toward oral health during prenatal care significantly influence patient outcomes; therefore, educational efforts targeting healthcare providers are crucial to improving prenatal oral health [6].

In France, the midwives perform medical monitoring of the pregnancy. Midwives monitor labor and delivery independently and, after birth, provide care for the mother and child. Midwifery education takes 6 years, combining theoretical and practical lessons, enabling student midwives to acquire the basics of obstetric, gynecological, and pediatric physiology. As pregnant women are frequently monitored during pregnancy by gynecologists and midwives, several recent studies investigated their level of knowledge to evaluate if oral health is considered during the routine visits of pregnant women [7, 11–13].

This study aimed to evaluate midwifery students’ knowledge level in France and determine whether they know the relationship between oral health and pregnancy.

## 2. Materials and Methods

### 2.1. Studied Population

This study was carried out using a self‐administered structured questionnaire. A questionnaire was emailed using Google Forms to the French students in midwifery (between 2nd and 5th year) in November 2023. The study protocol was approved by the Ethical Committee of the Faculty of Medicine (CE‐2025‐19). Participants were requested to complete anonymously the questionnaire. The study’s objectives were briefly explained at the beginning of the form. The questionnaire included 31 questions and was pretested to validate understandability and to apply required modifications when needed. The questionnaire included four domains. The first domain was related to the demographic characteristics of the participants. The second domain evaluated participants’ knowledge regarding the link between oral health and pregnancy. The third domain was assessing the consideration of oral health during their studies. The final domain aimed to evaluate their clinical experience related to oral health in pregnant women. Questionnaires included yes/no and multiple‐choice questions. About 10–15 min were necessary to complete the questionnaire.

### 2.2. Statistical Analysis

Analyses were performed using R software with the GMRC Shiny Stats package [14]. Univariate statistical analyses were performed to assess if there are potential correlations between answers. Proportions were compared using the chi‐squared and Fisher exact tests as appropriate for sample size.

## 3. Results

### 3.1. Population

A total of 420 students replied to the questionnaire. In this sample population, 21% of the participants were in the second year of their study, 19% in the third year, 29% in the fourth year, and 31% in the fifth year (Table 1). Interestingly, 17 students (4%) have children, and among them, one‐third (6/17) faced dental problems during their pregnancy.

**Table 1:** Distribution of the students by academic year of midwifery studies.

Study’s year	*n* (%)
2^nd^ year	89 (21.2)
3^rd^ year	79 (18.8)
4^th^ year	121 (28.8)
5^th^ year	130 (31)
Other (master program, …)	1 (0.2)

### 3.2. Link Between Oral Health and Pregnancy

Regarding students’ knowledge related to the link between oral health and pregnancy, 0.5% (2) considered it very good, 16% (67) as good, 16% (66) as nonexistent, and 67% (285) as insufficient. Indeed, only 16 were explicitly aware of the link between bad oral hygiene and/or periodontal diseases and adverse pregnancy outcomes. The influence of bacterial dissemination was the most cited potential cause (86%), followed by the systemic release of inflammatory cytokines from the periodontally compromised sites (78%). However, false beliefs were also cited by a significant number of participants (i.e., anxiety, pain) (Table 2).

**Table 2:** Knowledge of the link between oral health and pregnancy and of the causes of periodontal diseases in pregnancy.

Question	*n* (%)
How would you assess your current knowledge of the possible link between dental problems and pregnancy?	
Very good	2 (0.5)
Good	67 (16.4)
Nonexistent	66 (16.2)
Insufficient	285 (66.9)
What mechanisms explain the link between poor oral hygiene, periodontal diseases, and adverse pregnancy outcomes?	
Bacterial dissemination	363 (86)
Systemic release of inflammatory cytokines from the periodontal sites	329 (78)
Anxiety	106 (25)
Pain	116 (27)
Fatigue	144 (34)
No answer	3 (0.01)

Interestingly, only 51% of the students were aware of periodontitis, and those who feel more aware of the link between dental problems and pregnancy were more likely to know periodontitis (*p* < 0.01) (Table 3).

**Table 3:** Analysis of factors associated with the link between oral health and pregnancy, the education and the clinical application of the students’ knowledge.

Question	Answers		Explanatory variable	Answers		*p* value
	Yes	No		Yes	No	
Do you have kids?	17	403	Are the prevention and treatment of oral diseases important during pregnancy?	399	21	0.33
			Are you aware of the impact of poor oral hygiene and periodontal diseases on certain adverse pregnancy outcomes?	281	139	0.45
			Do you think that dental follow‐up and treatments are important during pregnancy?	408	12	0.47
			Do you know what periodontitis is?	214	206	0.51
			Do you think a pregnant woman is more likely to develop gum problems?	395	25	0.29

Question	Answers				Explanatory variable	Answers		*p* value
	Good	Nonexistent	Insufficient	Very good		Yes	No	
How would you assess your current knowledge of the possible link between dental problems and pregnancy?	67	66	285	2	Do you have kids?	17	403	0.14
					Are the prevention and treatment of oral diseases important during pregnancy?	399	21	<0.01
					Are you aware of the impact of poor oral hygiene and periodontal diseases on certain adverse pregnancy outcomes?	281	139	<0.01
					Do you think that dental follow‐up and treatments are important during pregnancy?	408	12	1
					Do you know what periodontitis is?	214	206	<0.01
					Do you think a pregnant woman is more likely to develop gum problems?	395	25	<0.01
					Have you ever covered the topic of periodontal diseases in your courses?	179	241	<0.01

Question	Answers				Explanatory variable	Answers			*p* value
	Good	Nonexistent	Insufficient	Very good		Yes	No	∅	
How would you assess your current knowledge of the possible link between dental problems and pregnancy?	67	66	285	2	Did you know that pregnant women can benefit, from the fourth month of pregnancy, from a dental prevention check‐up fully covered by health insurance?	207	53	160	<0.01

Question	Yes	No	Explanatory variable	Yes	No	*p* value
Have you ever covered the topic of periodontal diseases in your courses?	179	241	Are the prevention and treatment of oral diseases important during pregnancy?	399	21	0.07
			Are you aware of the impact of poor oral hygiene and periodontal diseases on certain adverse pregnancy outcomes?	281	139	<0.01
			Do you think that dental follow‐up and treatments are important during pregnancy?	408	12	0.6
			Do you know what periodontitis is?	214	206	<0.01
			Do you think a pregnant woman is more likely to develop gum problems?	395	25	<0.01

Question	Answers					Explanatory variable	Answers		*p* value
	Never	Sometimes	∅	Always	Often		Yes	No	
Do you advise your patients to have a dental check‐up during their pregnancy?	88	102	163	2	42	Are the prevention and treatment of oral diseases important during pregnancy?	399	21	0.45
						Are you aware of the impact of poor oral hygiene and periodontal diseases on certain adverse pregnancy outcomes?	281	139	0.02
						Do you think that dental follow‐up and treatments are important during pregnancy?	408	12	0.70
						Do you know what periodontitis is?	214	206	0.05
						Do you think a pregnant woman is more likely to develop gum problems?	395	25	0.78

Question	Answers			Explanatory variable	Answers				*p* value
	Yes	No	∅		Good	Non existent	Insufficient	Very good	
Have you ever encountered a dental problem with a patient or received a question from her about it?	70	186	164	How would you assess your current knowledge of the possible link between dental problems and pregnancy?	67	66	285	2	0.79
					**Yes**		**No**		
				Are the prevention and treatment of oral diseases important during pregnancy?	399		21		0.85
				Are you aware of the impact of poor oral hygiene and periodontal diseases on certain adverse pregnancy outcomes?	281		139		0.99
				Do you think that dental follow‐up and treatments are important during pregnancy?	408		12		0.95
				Do you know what periodontitis is?	214		206		0.63
				Do you think a pregnant woman is more likely to develop gum problems?	395		25		0.7

Question	Answers					Explanatory variable	Answers					*p* value
	Never	Sometimes	∅	Always	Often		Yes	No	∅	Rather yes	Rather no	
Do you advise your patients to have a dental check‐up during their pregnancy?	88	102	163	2	42	Do you feel comfortable giving oral hygiene advice (duration, frequency, brushing technique, interdental hygiene…)?	10	48	164	112	86	<0.01

Question	Answers					Explanatory variable	Answers				*p* value
	Yes	Rather yes	∅	Rather no	No		Good	Nonexistent	Insufficient	Very good	
Do you feel comfortable giving oral hygiene advice (duration, frequency, brushing technique, interdental hygiene…)?	10	112	164	86	48	How would you assess your current knowledge of the possible link between dental problems and pregnancy?	67	66	285	2	<0.01
							**Yes**		**No**		
						Are the prevention and treatment of oral diseases important during pregnancy?	399		21		0.86
						Are you aware of the impact of poor oral hygiene and periodontal diseases on certain adverse pregnancy outcomes?	281		139		0.31
						Do you think that dental follow‐up and treatments are important during pregnancy?	408		12		0.31
						Do you know what periodontitis is?	214		206		<0.01
						Do you think a pregnant woman is more likely to develop gum problems?	395		25		0.37

*Note:* The *p* value indicates the statistical significance of the association between the question of interest (left) and each explanatory variable (right).

Regarding the awareness of gingival and dental pathologies as risk factors for pregnancy complications, only 8% of respondents recognized gingival diseases as a potential risk. In comparison, 51% acknowledged the impact of dental issues. Interestingly, 83% believed that pregnancy is causing physiological changes in the gingiva, and 60% believed that pregnancy promotes the development of dental caries (Table 4).

**Table 4:** Awareness of gingival and dental pathologies as risk factors for pregnancy complications.

Question	*n* (%)
Gingival diseases as a potential risk	
Yes	36 (8)
No	384 (92)
Impact of dental issues	
Yes	217 (51)
No	203 (49)
Pregnancy causes pathological changes in gingiva	
Yes	351 (83)
No	69 (17)
Pregnancy promotes the development of dental caries	
Yes	252 (60)
No	168 (40)

Among symptoms related to potential periodontal disease, bleeding gums were the most recognized sign (90%), followed by mobile teeth (84%), discoloration of the teeth (64%), and halitosis (60%). Other indicators, such as dental plaque/calculus (34%), were less frequently cited.

Concerning common oral health conditions, during pregnancy, 92% identified gingivitis or periodontitis as a primary issue, whereas caries was recognized by 56%. Dental abscesses (90%) and oral ulcers (82%) were also frequently noted. Food impaction was mentioned by 20% of the participants, reflecting some awareness of less severe concerns. When assessing knowledge of oral health’s systemic impact during pregnancy, 65% were aware of its association with premature birth and only 17% with preeclampsia. Other complications, such as premature rupture of membranes (47%) and chorioamnionitis (58%), were frequently recognized.

### 3.3. Education

Regarding their education, 94% of the students reported having received oral health‐related lectures. However, when asked if they had ever covered the topic of periodontal diseases in their courses, only 43% of respondents confirmed doing so, indicating moderate integration of this subject into their curriculum. Those individuals were more likely to be aware of the impact of poor oral hygiene on pregnancy outcomes, recognize periodontitis, and believe that pregnant women are more susceptible to gum issues. Additionally, those with prior education on periodontal diseases demonstrated a stronger understanding of these associations (*p* < 0.01) (Table 3).

Interestingly, regarding the time spent learning about periodontal diseases, 34% of participants reported spending less than 2 h, 14% allocated 2–5 h, and only 3% over 5 h. Those who have spent more hours learning about the topic were more likely to recognize the importance of preventing and treating oral diseases, understand the impact of poor oral hygiene on pregnancy outcomes, know what periodontitis is, and believe that pregnant women are more prone to gum problems (*p* < 0.05).

### 3.4. Clinical Application

In total, 405 students (96%) were aware of the possibility of dental consultation during pregnancy. Overall, 95% considered dental follow‐up and dental care essential during pregnancy. Regarding providing oral hygiene advice during midwifery consultations, only 2% of respondents reported consistently counseling pregnant patients on maintaining proper oral hygiene. Notably, 49% of participants knew pregnant women could access a fully covered oral health prevention exam starting from the fourth month of pregnancy. Despite a modest increase, the percentage remains prominently low (16%) for those who encouraged frequently their patients to schedule dental check‐ups during pregnancy, underscoring the need for preventive measures (Table 5). Those encouraging check‐ups may also feel more confident in guiding their patients (*p* < 0.01) (Table 3).

**Table 5:** Practice and confidence in oral health promotion during pregnancy.

Clinical practice	*n* (%)
Providing oral hygiene advice during midwifery consultations	
Never	158 (38)
Sometimes	91 (21)
Always	3 (1)
Often	4 (1)
∅	164 (39)
Encouraged their patients to schedule dental check‐ups during pregnancy	
Never	88 (21)
Sometimes	102 (24)
Always	25 (6)
Often	42 (10)
∅	163 (39)
Realizing oral examination for hospitalized patients with pregnancy or delivery risks	
Never	231 (55)
Sometimes	21 (5)
Often	2 (0.5)
Always	0 (0)
∅	166 (39,5)
Confidence in offering oral hygiene instructions	
Yes	10 (2)
Mostly yes	86 (20)
Mostly no	112 (28)
No	48 (11)
∅	164 (39)

For patients at risk of adverse outcomes, 55% had never conducted oral examinations to evaluate potential infection risks, reflecting limited integration of oral health screening in these high‐risk cases (Table 5). Regarding confidence in offering oral hygiene guidance, including brushing duration, frequency, technique, and interdental care, 23% felt well‐prepared, while a significant proportion (38%) expressed uncertainty in their abilities (Table 5). Students who felt more comfortable offering oral hygiene guidance were likelier to report a higher level of knowledge about the relationship between dental issues and pregnancy outcomes (*p* < 0.01) (Table 3).

## 4. Discussion

This study aimed to assess the perception of midwifery students about oral health during pregnancy, as pregnant women are more at risk of oral diseases during this particular period of life [1].

In previous surveys, many obstetricians and gynecologists in France demonstrated knowledge of the relationship between periodontal diseases and pregnancy outcomes [11]. Conversely, there was a noticeable gap between their awareness and clinical practice [15]. This aligns with findings from previous studies, where oral health was not always prioritized during consultations [11, 16, 17]. Similarly, the practitioners with more experience and those with personal dental histories were more likely to refer patients to dental care. These gaps reinforce the need for ongoing professional development and systemic changes to ensure that oral health is adequately addressed within the scope of maternity care [11].

Midwives play a crucial role in addressing these issues and encouraging dental visits. This supports the notion that midwives can effectively advocate for oral health during pregnancy, facilitating a more holistic care approach [18]. Research suggests that pregnant women are generally aware of oral health, but the specific risks related to pregnancy remain unclear [4, 5]. Another study highlights the significance of successful periodontal therapy in reducing the risk of preterm birth, suggesting that effective treatment can improve pregnancy outcomes, particularly for women with periodontal disease [19]. This aligns with findings in other research suggesting that although nonsurgical periodontal therapy during pregnancy may not constantly improve pregnancy outcomes, it does promote general health and improves the overall oral condition of the patient, potentially reducing risks linked to maternal and fetal health [9].

In this study, the participants showed moderate awareness regarding the systemic impact of oral health during pregnancy. This suggests that although the students recognized some key systemic risks tied to poor oral health, they might not fully grasp the broader range of complications that periodontal diseases can lead to during pregnancy. It was also shown in another study conducted in France that French health professionals, including midwives, demonstrate limited knowledge of oral health’s impact on pregnancy, emphasizing the need for interdisciplinary education to prevent adverse outcomes [12]. Considering the potential impacts on maternal and fetal health, this situation indicates that oral health should be more comprehensively included in prenatal care practices, particularly in midwifery education. Even though, in this study, there is a general recognition of the importance of oral health, only 43% of respondents indicated that periodontal diseases were covered during their training, and a mere 3% received more than 5 h of focused education on the topic. Similarly, a study in the United States found that although all midwifery programs addressed oral health, fewer than a quarter of the programs’ directors were satisfied with the competency of their graduates in this area [20]. These findings reveal that although oral health is included in the curriculum, there is a lack of emphasis on periodontal diseases, especially compared to other obstetric and gynecological subjects [20]. An Australian initiative designed a dedicated oral health module for midwifery students, incorporating blended learning approaches, which improved knowledge of oral health practices during pregnancy [21]. This model can serve as a valuable template for enhancing oral health education in midwifery programs globally [21]. Another French study found that although health professionals, including midwives, recognize the importance of oral health during pregnancy, only dentists reported receiving adequate training on this topic [2]. The study highlights the need for improved oral health education for midwives to address gaps in prenatal care [2]. Similar trends have been observed in Central‐Eastern Europe, where midwifery education models possess a relatively strong theoretical component but lack sufficient practical training [22]. This underscores the need for a balanced curriculum prioritizing theoretical knowledge and practical skills [22]. In France, midwifery education follows a rigorous, structured program that includes various aspects of prenatal, delivery, and postnatal care. Despite this comprehensive approach, oral health education remains insufficiently emphasized, indicating the need for a more holistic approach to maternal care [23].

This study also highlights a concerning trend: as students advance in their studies, their depth of knowledge in periodontal education declines. This is particularly concerning because the later years are vital for solidifying the knowledge and skills necessary for comprehensive maternal care [24]. A similar issue is observed in a recent study in France, where midwives report insufficient training on oral health, particularly during pregnancy. This is understandable, as oral health education is not traditionally a core component of gynecological or midwifery training. However, with only 30% of midwives having received relevant training during their initial education, there may be a need to enhance interdisciplinary collaboration to bridge this gap [15].

Furthermore, studies have shown that midwives and nurses are in an ideal position to promote oral health in maternal and child care. This emphasizes the potential role of midwives in preventive oral care and screening, especially given the gaps in current education and practice regarding oral health among midwifery students [15]. Enhancing midwifery education with oral health training can equip students to offer both education and practical care, improving health outcomes for both mothers and children [25].

This study highlights a concerning issue as only 2% of students actively guide maintaining oral hygiene for pregnant women. Even with an understanding of oral health’s significance, midwifery students are not sufficiently equipped to give practical advice on preventive oral care, which is essential during pregnancy. Innovative educational tools like mind maps could enhance midwifery students’ understanding of oral health. Such techniques have improved information retention and critical thinking in healthcare education, offering a potential strategy for improving oral health coverage during pregnancy [26]. Moreover, 55% of students admitted they do not conduct oral health screenings for hospitalized pregnant women, suggesting that oral health is not consistently prioritized in the management of high‐risk pregnancies.

Even though this study offers important insights into the current state of oral health education among midwifery students, it is essential to recognize some limitations. The reliance on self‐reported questionnaires may introduce bias, and the study’s cross‐sectional design restricts the ability to track changes in knowledge over time. Furthermore, the sample was limited to midwifery students in France, so the findings may not be relevant to other regions or healthcare professionals. Furthermore, some parameters were not considered in the questionnaire. Indeed, to avoid bias related to anonymity, we decided to avoid questions related to participant’s gender, as there is a low number of male students. Nevertheless, consideration of the self‐perceived importance of oral and periodontal health of the participants was not assessed. Future research should aim to enhance the integration of oral health topics within midwifery curricula, ensuring that students are well‐informed to address the oral health needs of pregnant women and addressing the imbalance between theoretical and practical training, as noted in midwifery education systems in Central‐Eastern Europe [22]. Additionally, clinical practice guidelines should promote routine oral health evaluation during pregnancy, especially for high‐risk patients. Public health initiatives should also prioritize educating pregnant women about the significance of oral health, ensuring they have access to accurate and easily understandable information to facilitate timely dental care.

## 5. Conclusions

This study underlined that there are still gaps in the knowledge and practice of oral health care during pregnancy among midwifery students. Although most of the participants agreed that oral health is an important issue during pregnancy, they did not fully comprehend the level of association between periodontal diseases and pregnancy complications. Moreover, there was a discrepancy between students’ knowledge and their capacity to give practical oral health advice to pregnant women. This calls for additional and more detailed training in this field.

## Author Contributions

**Aikaterini Vasileiou**: formal analysis, writing – original draft. **Lucie Brillaux**: conceptualization, investigation, writing – original draft. **Catherine Petit**: visualization, writing – review and editing. **Pierre-Yves Gegout**: visualization, writing – review. **Olivier Huck**: conceptualization, formal analysis, methodology, supervision, validation, writing – original draft.

## Funding

The authors have nothing to report.

## Ethics Statement

The study protocol was approved by the Ethical Committee of the Faculty of Medicine (CE‐2025‐19).

## Conflicts of Interest

The authors declare no conflicts of interest.

## Data Availability

The data that support the findings of this study are available from the corresponding author upon reasonable request.

## References

[1] Ye C. and Kapila Y. , Oral Microbiome Shifts During Pregnancy and Adverse Pregnancy Outcomes: Hormonal and Immunologic Changes at Play, Periodontology 2000. (2021) 87, no. 1, 276–281, 10.1111/prd.12386. 34463984 PMC8457099

[2] Egea L. , Le Borgne H. , Samson M. , Boutigny H. , Philippe H.-J. , and Soueidan A. , Infections Buccodentaires et Complications de la Grossesse: Connaissances et Attitudes Des Professionnels de Santé, Gynécologie Obstétrique & Fertilité. (2013) 41, no. 11, 635–640, 10.1016/j.gyobfe.2012.09.007, 2-s2.0-84888204411. 23602137

[3] Wu M. , Chen S.-W. , and Jiang S.-Y. , Relationship Between Gingival Inflammation and Pregnancy, Mediators of Inflammation. (2015) 2015, no. 1, 10.1155/2015/623427, 2-s2.0-84927142927, 623427. 25873767 PMC4385665

[4] Schröter U. , Ziebolz D. , Stepan H. , and Schmalz G. , Oral Hygiene and Oral Health Behavior, Periodontal Complaints and Oral Health-Related Quality of Life in Pregnant Women, BMC Oral Health. (2022) 22, no. 1, 10.1186/s12903-022-02508-4, 476. 36348335 PMC9644595

[5] Daalderop L. A. , Wieland B. V. , and Tomsin K. , et al.Periodontal Disease and Pregnancy Outcomes: Overview of Systematic Reviews, JDR Clinical & Translational Research. (2018) 3, no. 1, 10–27, 10.1177/2380084417731097, 2-s2.0-85048835879. 30370334 PMC6191679

[6] Huck O. , Tenenbaum H. , and Davideau J.-L. , Relationship Between Periodontal Diseases and Preterm Birth: Recent Epidemiological and Biological Data, Journal of Pregnancy. (2011) 2011, 10.1155/2011/164654, 164654. 22132334 PMC3205685

[7] Bechina C. , Bonvillain G. , and Rethore G. , et al.Knowledge and Practice Behaviours of Obstetricians/Gynecologists and Midwives Concerning Periodontal Health and Pregnancy, Oral Health and Preventive Dentistry. (2023) 21, b4586823. 37916549 10.3290/j.ohpd.b4586823 PMC11653776

[8] Sanz M. , Herrera D. , and Kebschull M. , et al.Treatment of Stage I-III Periodontitis-the EFP S3 Level Clinical Practice Guideline, Journal of Clinical Periodontology. (2020) 47, no. Suppl 22, 4–60. 32383274 10.1111/jcpe.13290 PMC7891343

[9] Bobetsis Y. A. , Ide M. , Gürsoy M. , and Madianos P. N. , Periodontal Diseases and Adverse Pregnancy Outcomes. Present and Future, Periodontology 2000. (2023) 1–28, 10.1111/prd.12486. 37149740

[10] Kamalabadi Y. M. , Campbell M. K. , Zitoun N. M. , and Jessani A. , Unfavourable Beliefs About Oral Health and Safety of Dental Care During Pregnancy: A Systematic Review, BMC Oral Health. (2023) 23, no. 1, 10.1186/s12903-023-03439-4, 762. 37840149 PMC10577919

[11] Cohen L. , Schaeffer M. , and Davideau J. , et al.Obstetric Knowledge, Attitude, and Behavior Concerning Periodontal Diseases and Treatment Needs in Pregnancy: Influencing Factors in France, Journal of Periodontology. (2015) 86, no. 3, 398–405, 10.1902/jop.2014.140371, 2-s2.0-84924942557. 25427617

[12] Boutigny H. , de Moegen M.-L. , and Egea L. , et al.Oral Infections and Pregnancy: Knowledge of Gynecologists/Obstetricians, Midwives and Dentists, Oral Health & Preventive Dentistry. (2016) 14, no. 1, 41–47, 10.3290/j.ohpd.a34376, 2-s2.0-84973408771. 26106653

[13] Liu Y. , Li T. , and Guo N. , et al.Women’s Experience and Satisfaction With Midwife-Led Maternity Care: A Cross-Sectional Survey in China, BMC Pregnancy and Childbirth. (2021) 21, no. 1, 10.1186/s12884-021-03638-3, 151. 33607963 PMC7893951

[14] Fabacher T. , Schaeffer M. , and Tuzin N. , et al.[Medical Biostatistics With GMRC Shiny Stats - Learning by Doing], Annales Pharmaceutiques Françaises. (2020) 78, no. 6, 499–506, 10.1016/j.pharma.2020.06.001. 32565157

[15] Bossouf A. , Sabourin C. , Fuchs F. , Giraudeau N. , and Inquimbert C. , Interprofessional Survey on Knowledge and Attitudes of Midwives Regarding Oral Health, in France, European Journal of Midwifery. (2023) 7, 10.18332/ejm/172881, 37. 38045473 PMC10690821

[16] Owens J. B. , Wilder R. S. , Southerland J. H. , Buse J. B. , and Malone R. M. , North Carolina Internists’ and Endocrinologists’ Knowledge, Opinions, and Behaviors Regarding Periodontal Disease and Diabetes: Need and Opportunity for Interprofessional Education, Journal of Dental Education. (2011) 75, no. 3, 329–338, 10.1002/j.0022-0337.2011.75.3.tb05046.x. 21368257

[17] Zanata R. L. , Fernandes K. B. P. , and Navarro P. S. L. , Prenatal Dental Care: Evaluation of Professional Knowledge of Obstetricians and Dentists in the Cities of Londrina/PR and Bauru/SP, Brazil, 2004, Journal of Applied Oral Science. (2008) 16, 194–200. 19089217 10.1590/S1678-77572008000300006 PMC4327693

[18] Petit C. , Benezech J. , and Davideau J.-L. , et al.Consideration of Oral Health and Periodontal Diseases During Pregnancy: Knowledge and Behaviour Among French Pregnant Women, Oral Health and Preventive Dentistry. (2021) 19, 33–42. 33491376 10.3290/j.ohpd.b875513 PMC11641336

[19] Jeffcoat M. , Parry S. , Sammel M. , Clothier B. , Catlin A. , and Macones G. , Periodontal Infection and Preterm Birth: Successful Periodontal Therapy Reduces the Risk of Preterm Birth, BJOG: An International Journal of Obstetrics & Gynaecology. (2011) 118, no. 2, 250–256, 10.1111/j.1471-0528.2010.02713.x, 2-s2.0-78650357970. 20840689

[20] Haber J. , Dolce M. C. , and Hartnett E. , et al.Integrating Oral Health Curricula Into Midwifery Graduate Programs: Results of a US Survey, Journal of Midwifery & Women’s Health. (2019) 64, no. 4, 462–471, 10.1111/jmwh.12974, 2-s2.0-85065089957. 31034757

[21] Duff M. , Dahlen H. G. , Burns E. , Priddis H. , Schmied V. , and George A. , Designing an Oral Health Module for the Bachelor of Midwifery Program at an Australian University, Nurse Education in Practice. (2017) 23, 76–81, 10.1016/j.nepr.2017.02.005, 2-s2.0-85014424004. 28273559

[22] Mivšek P. , Baškova M. , and Wilhelmova R. , Midwifery Education in Central-Eastern Europe, Midwifery. (2016) 33, 43–45, 10.1016/j.midw.2015.10.016, 2-s2.0-84949646889. 26632483

[23] Forestier R. , Midwifery in France, Journal of Nurse-Midwifery. (1983) 28, no. 4, 37–38, 10.1016/0091-2182(83)90109-X, 2-s2.0-0020788246. 6554314

[24] Marquès-Pellejà G. , Roqueta-Vall-Llosera M. , and Cámara-Liebana D. , et al.Assessing the Student Nurses’ Knowledge of Oral Health Care, Nursing Reports. (2023) 13, no. 3, 1126–1137, 10.3390/nursrep13030097. 37606465 PMC10443348

[25] Abou El Fadl R. , Blair M. , Hassounah S. , and van Wouwe J. , Integrating Maternal and Children’s Oral Health Promotion Into Nursing and Midwifery Practice–A Systematic Review, PLoS ONE. (2016) 11, no. 11, 10.1371/journal.pone.0166760, 2-s2.0-84996497363. PMC5120808 27880790

[26] Noonan M. , Mind Maps: Enhancing Midwifery Education, Nurse Education Today. (2013) 33, no. 8, 847–852, 10.1016/j.nedt.2012.02.003, 2-s2.0-84881022840. 22386316

